# Differentiation of Recurrence from Radiation Necrosis in Gliomas Based on the Radiomics of Combinational Features and Multimodality MRI Images

**DOI:** 10.1155/2019/2893043

**Published:** 2019-12-01

**Authors:** Quan Zhang, Jianyun Cao, Junde Zhang, Junguo Bu, Yuwei Yu, Yujing Tan, Qianjin Feng, Meiyan Huang

**Affiliations:** ^1^Guangdong Provincial Key Laboratory of Medical Image Processing, School of Biomedical Engineering, Southern Medical University, Guangzhou, China; ^2^Zhujiang Hospital, Southern Medical University, Guangzhou, China

## Abstract

**Purpose:**

To classify radiation necrosis versus recurrence in glioma patients using a radiomics model based on combinational features and multimodality MRI images.

**Methods:**

Fifty-one glioma patients who underwent radiation treatments after surgery were enrolled in this study. Sixteen patients revealed radiation necrosis while 35 patients showed tumor recurrence during the follow-up period. After treatment, all patients underwent T1-weighted, T1-weighted postcontrast, T2-weighted, and fluid-attenuated inversion recovery scans. A total of 41,284 handcrafted and 24,576 deep features were extracted for each patient. The 0.623 + bootstrap method and the area under the curve (denoted as 0.632 + bootstrap AUC) metric were used to select the features. The stepwise forward method was applied to construct 10 logistic regression models based on different combinations of image features.

**Results:**

For handcrafted features on multimodality MRI, model 7 with seven features yielded the highest AUC of 0.9624, sensitivity of 0.8497, and specificity of 0.9083 in the validation set. These values were higher than the accuracy of using handcrafted features on single-modality MRI (paired *t*-test, *p* < 0.05, except sensitivity). For combined handcrafted and AlexNet features on multimodality MRI, model 6 with six features achieved the highest AUC of 0.9982, sensitivity of 0.9941, and specificity of 0.9755 in the validation set. These values were higher than the accuracy of using handcrafted features on multimodality MRI (paired *t*-test, *p* < 0.05).

**Conclusions:**

Handcrafted and deep features extracted from multimodality MRI images reflecting the heterogeneity of gliomas can provide useful information for glioma necrosis/recurrence classification.

## 1. Introduction

Gliomas are the most common and aggressive brain tumors in adults and have an approximate 5-year survival rate of 10% in their highest grade (e.g., glioblastoma multiforme) [1]. The conventional therapy for gliomas is surgery followed by conventional radiotherapy/chemotherapy [1, 2]. However, this combinatory therapy usually leads to radiation necrosis, which is the most common side effect in gliomas within 2 years after treatment [3, 4]. Unfortunately, the period of occurrence of radiation necrosis is also the peak period of glioma recurrence [4]. Clinically, the methods used to distinguish between glioma recurrence and necrosis are follow-up, biopsy, and surgical resection [5]. Given that the treatment protocols of glioma necrosis and recurrence are quite different [6, 7], finding a fast and noninvasive way to differentiate glioma necrosis from recurrence is important.

Radiomics [8] is widely used as a noninvasive method to classify lesions into recurrence or necrosis [9, 10]. In the current radiomics models, good classification results are achieved by using handcrafted features (e.g., intensity and texture features). However, handcrafted features are shallow and low-ordered; as such, they may not fully characterize tumor heterogeneity and, in fact, could limit the potential of the radiomics model applied [11]. To solve this problem, a number of studies have proposed the use of deep features [12–15]. In these studies, improvements in performance were observed by incorporating deep features into the radiomics model of interest. Thus, exploiting potential tumor heterogeneity by using deep features is expected to provide a new and effective point of view from which to improve glioma necrosis and recurrence classification.

**Figure 1:**
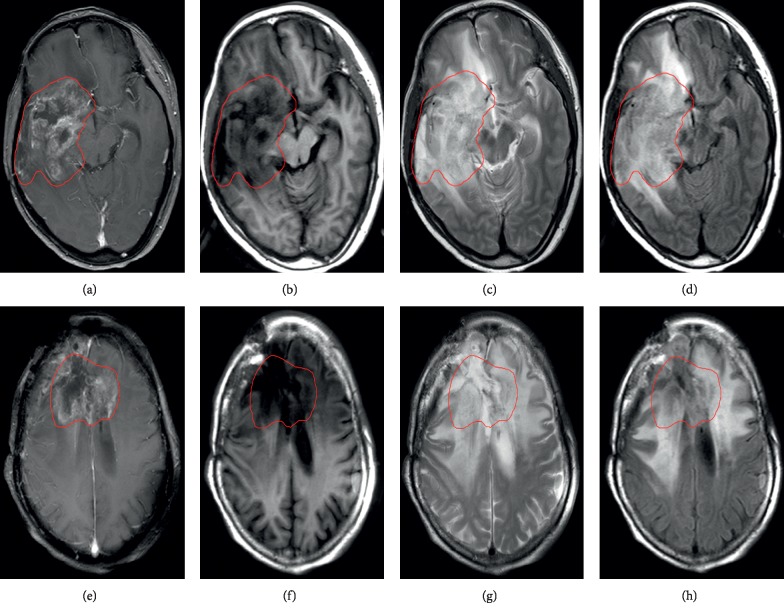
MRI diagnostic images of two patients with glioma. (a–d) Recurrent image of a 52-year-old patient with astrocytoma 1 year after radiotherapy. (e–h) Necrotic image of a 54-year-old patient with oligodendroglioma 6 months after radiotherapy. (a), (b), (c), and (d) and (e), (f), (g), and (h), respectively, show T1C, T1, T2, and FLAIR images. The inside of the red line shows the edge of the lesion.

Frequent monitoring is required for cancer patients; among the imaging methods available, MRI is consistently the preferred technique [16]. Different MRI modalities, such as magnetic resonance spectroscopy [17, 18], T1-weighted postcontrast (T1C) imaging [19], and diffusion-weighted imaging [20], are used to differentiate glioma necrosis from recurrence. However, most previous studies employ image information from single-modality MRI. Moreover, during follow-up, the most commonly used scans for glioma patients are T1-weighted (T1), T1C, T2-weighted (T2), and fluid-attenuated inversion recovery (FLAIR) images. Single-modality MRI provides partial information, whereas multimodality MRI comprehensively characterizes tissues [21, 22]. Therefore, combining different MRI modalities can enhance the tumor discriminatory power of the technology and reveal the degree of tumor infiltration [21, 23, 24].  Figure 1 shows that the heterogeneities of glioma recurrence and necrosis in different MRI modalities are different.

In this study, we proposed a novel radiomics model for distinguishing necrosis versus glioma recurrence. The contributions of our study were as follows. First, multimodality MRI images were used in this research. Different MRI modalities could reveal different parts of the tumor area [22]. Therefore, the accuracy of glioma identification could be improved by using multimodality MRI images [25]. Second, deep features were combined with handcrafted features in this study to classify glioma necrosis versus recurrence. The powerful ability of deep features has been verified in previous studies [13–15]. Moreover, to the best of our knowledge, previous studies have not combined multimodality MRI images and deep features for classifying glioma necrosis versus recurrence. Therefore, the proposed method might be a valuable tool for distinguishing glioma necrosis from recurrence.

## 2. Materials and Methods

### 2.1. Study Population and MRI Images

This retrospective study was supported by the ethics committee of hospital and written informed consent was waived.

In this study, the diagnosis of glioma recurrence and necrosis was confirmed by two neuroradiologists with work experiences of over 9 and 20 years. Patients were included on the basis of the following criteria: (1) pathologically confirmed that glioma recurrence or necrosis occurred after radiotherapy; (2) all glioma patients' recurrence or necrosis after radiotherapy was confirmed by imaging and clinical follow-up (follow-up time > 6 months); (3) all MRI images (T1, T1C, T2, and FLAIR images) of glioma necrosis and recurrence used must be confirmed at a follow-up of no less than 6 months after radiotherapy; and (4) glioma patients without pathologic diagnosis excluded the possibility of pseudoprogression based on follow-up. If the follow-up time was not enough, it was difficult for the neuroradiologist to judge whether the patient has recurrence or pseudoprogression. Such glioma patients were not accepted. The exclusion criteria were as follows: (1) patients with recurrent glioma without radiotherapy; (2) follow-up time of less than 6 months for glioma patients; and (3) glioma patients without four modalities of MRI images. A total of 51 patients (16 necrosis and 35 recurrences) were enrolled in this study. The clinical characteristics of all patients are summarized in Table 1.

MRI images were obtained by using 3.0 T MRI machines (Philips, Achieva). The MRI protocols for the four modalities are listed in Table 2. All MRI scans were obtained in the axial plane.  Figure 1 illustrates an example of the four modalities of MRI images of glioma recurrence and necrosis.

### 2.2. Overview of the Proposed Method

**Figure 2:**
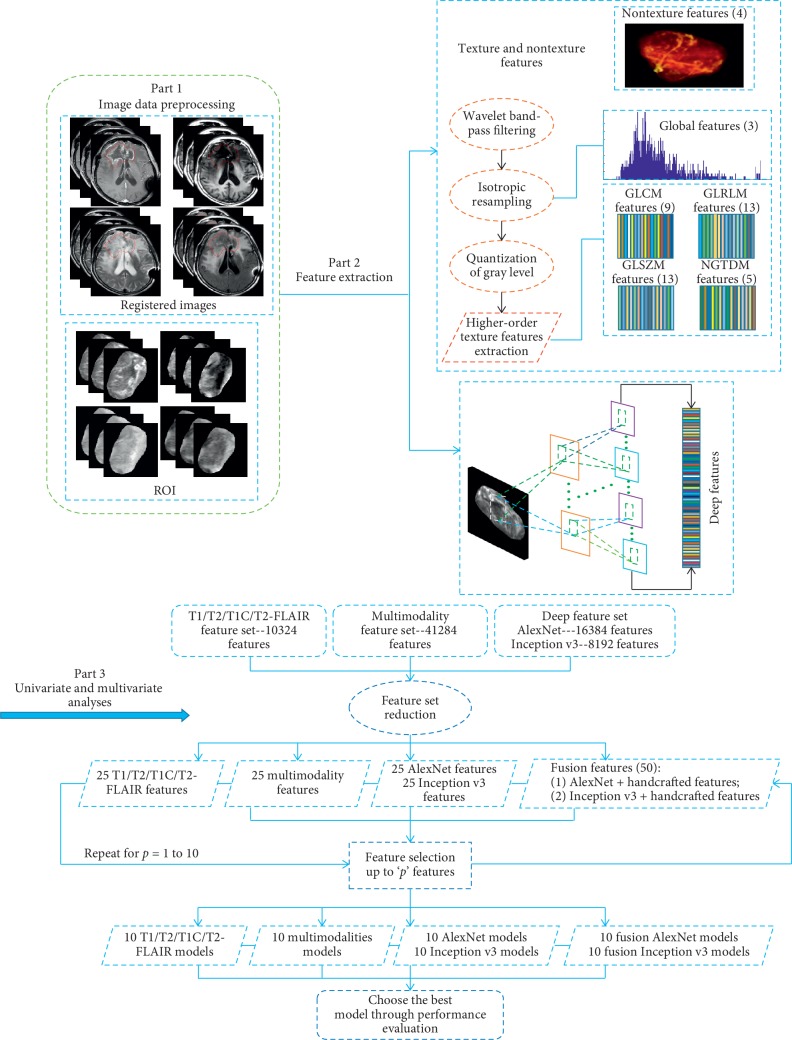
Overall framework of the proposed method.

The overall framework of the proposed method is shown in Figure 2. The method consists of three fundamental steps: (1) an image preprocessing step that obtains tumor regions, (2) a feature extraction step that extracts handcrafted and deep features, and (3) an analysis step that combines univariate and multivariate analyses. This combination allows the selection of features and construction of a prediction model for glioma necrosis and recurrence classification.

#### 2.2.1. Image Preprocessing

We used the linear registration function in FSL5.0.9 (http://fsl.fmrib.ox.ac.uk) to register T1, T2, and FLAIR images to T1C images and then applied ITK-SNAP software (http://www.itk-snap.org) to manually segment the tumor region for each patient. All manual segmentations of the tumor region were drawn slice-by-slice by a neuroradiologist with over 9 years of experience in neuroradiology. To avoid mistakes, another senior neuroradiologist with 20 years of experience in brain tumor diagnosis confirmed the final tumor region. All segmentations were drawn on T1C images, covered the entire tumor (avoid cystic changes, edema, and blood vessels), and used to extract handcrafted and deep features.

#### 2.2.2. Feature Extraction

The methodology used to extract handcrafted features from the tumor region and texture extraction parameters is described in the Supplementary Information. A total of 4 nontexture and 41,280 texture parameter features (10,320 features from each image of each MRI modality) were extracted for each patient.

We selected AlexNet [26] and Inception v3 [27], which were pretrained on approximately 1.2 million images from the ImageNet Dataset. Features were obtained by forward propagating an MRI slice through the network and extracting the deep features. The architectures of both networks are illustrated in the Supplementary Information. In our glioma dataset, the number of glioma lesion slices and the size of the glioma lesion area in each slice were different for different patients. If we used all slices of a glioma lesion for each patient, the feature dimension would be varied across different patients. Therefore, a 3D bounding box was used to extract the tumor region and then to extract the axial slice with the largest tumor area from this box. We also extracted the front and back slices of the extracted slice. Finally, the three axial slices were combined as an RGB volume. AlexNet and Inception v3 network were a continuous convolution and pooling operation on the input images. Compared with the shallower layer of the two networks, more heterogeneous information of tumors can be extracted in the deeper layer. Moreover, the penultimate layer of the deep network had been used to extract deep features in some recent studies [15, 28, 29], and good performance could be achieved, which indicated the effectiveness of the deep features extracted from the deeper layers. Therefore, for AlexNet, the RGB volume was converted into a size of 227 × 227 × 3 as the input, and the penultimate layer (FC7) was used as the output. A total of 16,384 AlexNet features (4,096 features from each image of each MRI modality) were found. For Inception v3, the RGB volume was adjusted to a size of 299 × 299 × 3 as the final input, and the average pooling layer was used as the output. Therefore, a total of 8,192 Inception v3 features (2048 features from each image of each MRI modality) were drawn.

#### 2.2.3. Univariate and Multivariable Analyses

Univariate associations between feature sets (4 nontexture features, 41,280 texture features, 16,384 AlexNet features, and 8,192 Inception v3 features) and glioma necrosis or recurrence were assessed using Spearman's rank correlation (*r*_*s*_). Given the existence of multiple comparisons, Bonferroni correction was also applied. The significance level was set to *p*=*α*/*K*, where *K* is the number of comparisons and *α* is the significance level set to 0.05.

In multivariate analysis, our goal is to find a linear combination of interesting features so that whether the output is necrosis or recurrence for the new input data could be properly judged. Therefore, the prediction model was constructed by using a logistic regression model:

(1) $$\begin{array}{c} y\left(x_{i}\right)=\overset{p}{\underset{j=1}{\sum }}a_{j}x_{ij}+a_{0},\;\text{for }i=1,2,\ldots N, \end{array}$$

where *x*_*ij*_ is the *j* th input variable (image features) of *i* th patient *x*_*i*_ and *a*={*a*_*j*_ ∈ *ℝ* : *j*=1,2,…, *p*} is the regression coefficient of the model for a total of *N* patients.

We employed the 0.623 + bootstrap method and the area under the curve (denoted as 0.632 + bootstrap AUC) metric to estimate which model learned from our dataset could best classify glioma recurrence and necrosis on a new sample. Before presenting the estimation method, we provide a brief symbol introduction. Let our image dataset be denoted as *x*={*x*_*i*_, *i*=1,2,…, *N*}. We construct a bootstrap sample *x*^*∗*^={*x*_*i*_^*∗*^ : *i*=1,2,…, *N*} with *N* patients randomly drawn with replacement from *x*. An original sample that does not appear in the bootstrap sample was defined as *x*^*∗*^(0). The generation of a large number *B* (*B* = 1000) of randomly drawn bootstrap samples *x*^*∗b*^ (*b* = 1, 2,…, *B*) is used to estimate a statistical quantity of interest on the unknown true population distribution. Note that the probability of selecting a positive (necrosis group class) is made equal to the probability of selecting a negative (recurrence group class) each time by drawing *x*^*∗b*^ from *x*; this approach is called “imbalance-adjusted bootstrap resampling.”

Prediction models were constructed for three different types of initial feature sets: (1) four sets of single-modality handcrafted features (T1, T1C, T2, and FLAIR), including 10,320 texture and 4 nontexture features for each modality; (2) multimodality handcrafted features, including 41,280 texture and 4 nontexture features; and (3) two sets of multimodality deep features, including 16,384 AlexNet features and 8,192 Inception v3 features. However, the number of feature sets being too large, many redundant and irrelevant features would cause overfitting. Therefore, feature reduction was applied to reduce feature dimensionality. We then used the 0.632 + bootstrap method AUC metric to select features to construct different orders of regression models. Finally, we selected the optimal model from the constructed models for classification. We provide details of each step in the following sections.

*(1) Feature Reduction*. The feature set reduction was performed by a stepwise forward feature selection scheme in order to create reduced feature sets containing 25 different features from larger initial sets, a procedure carried out using the gain equation:

(2) $$\begin{array}{c} {\bar{\text{Gain}}}_{j}=\gamma \cdot \left|{\hat{r}}_{s}\left(x_{j},y\right)\right|+\delta_{a}\left[\overset{d}{\underset{c=1}{\sum }}\left(\frac{2\left(d-c+1\right)}{d\left(d+1\right)}\right)\bar{\left(1-\text{MIC}\left(x_{c},x_{j}\right.\right)}\right]+\delta_{b}\cdot \left[\frac{1}{D}\overset{D}{\underset{k=1}{\sum }}\bar{\left(1-\text{MIC}\left(x_{k},x_{j}\right.\right)}\right], \end{array}$$

where ${\hat{r}}_{s}\left(x_{j},y\right)=\left(1/B\right)\sum_{b=1}^{B}r_{s}\left(x_{j}^{*b}y,\right)$ and $\bar{\left(1-\text{MIC}\left(x_{c},x_{j}\right.\right)}=\left(1/B\right)\sum_{b=1}^{B}\left(1-\text{MIC}\left(x_{c}^{*b},x_{j}^{*b}\right)\right)$.

In equation (2), *r*_*s*_(*x*_*j*_, *y*) is Spearman's rank correlation coefficient between each feature *j* and the output vector *y*={*y*_*i*_ ∈ {0 : recurrence, and 1 : necrosis} : *i*=1,2,…, *N*}, MIC(*x*_*c*_, *x*_*j*_) is the maximal information coefficient between features *c* and *j* [30], *d* is the selected feature for the reduced feature set, and *D* is the feature that has not been removed from the initial feature set. Parameters *γ*, *δ*_*a*_, and *δ*_*b*_ are set to 0.5, 0.5, and 0, respectively. During each iteration, a new feature with the largest gain value is selected for the reduced feature set, after which a new gain can be calculated based on the features reserved from the initial feature set using imbalance-adjusted bootstrap resampling (*B* = 1,000). The final selected 25 features are arranged in descending order according to their gain value because the gain equation uses *r*_s_ varying over the whole feature set.

*(2) Feature Selection*. After feature reduction, we obtained 25 features for each of the three initial feature sets. We then combined the deep features obtained from AlexNet and Inception v3 features with the handcrafted features from multimodality MRI images to obtain 50 fusion features (denoted as fusion AlexNet and fusion Inception v3 features). Using the reduced feature sets, stepwise forward feature selection was performed by maximizing the 0.632 + bootstrap AUC. The order of the regression model was set from 1 to 10, where the order value is the number of features to be selected. For a given model order, 25 independent experiments were conducted for each of the three initial feature sets and 50 independent experiments were performed for each of the two fusion feature sets. In each independent experiment, different features from the reduced set were assigned as a different “starter.” For each given starter, 1,000 logistic regression models were first created for the remaining features by using imbalance-adjusted bootstrap resampling. Then, the single feature maximizing the 0.632 + bootstrap AUC, defined in equation (3), was selected. This process was repeated up to order 10, after which the combination of features yielding the highest 0.632 + bootstrap AUC for each model was identified. To clarify, we used 1-order regression model as an example. For the 25 selected features, each of them could be utilized to construct a 1-order regression model, and thus, there were 25 1-order regression models. For a given 1-order regression model, we first resampled the sample 1000 times to get 1000 pairs of training sets and validation sets. Then, 1000 experiments were carried out and the average of AUC of 1000 experiments was obtained for the model. Therefore, for a 1-order regression model, we got 25 averaged AUC values. For the 2–10 order features, the same process was performed as above. Finally, we selected the order corresponding to the maximum averaged AUC value as the final feature combination for the classification of glioma necrosis versus recurrence.

(3) $$\begin{array}{c} {\bar{\left[\text{AUC}\right]}}_{0.632+}=\frac{1}{B}\overset{B}{\underset{b=1}{\sum }}\left[\left(1-\alpha \left(b\right)\right)\cdot \text{AUC}\left(x,x\right)+\alpha \left(b\right)\cdot {\text{AUC}}^{\prime }\left(x^{*b},x^{*b}\left(0\right)\right)\right], \end{array}$$

where

(4) $$\begin{array}{c} \text{AUC}\prime \left(x^{*b},x^{*b}\left(0\right)\right)=\max\left\{0.5,\text{AUC}\left(x^{*b},x^{*b}\left(0\right)\right)\right\},\alpha \left(b\right)=\frac{0.632}{1-0.368\cdot R\left(b\right)},\\ R\left(b\right)=\left\{\begin{array}{cc} 1,\text{if }\text{AUC}\left(x^{*b},x^{*b}\left(0\right)\right)<0.5,\\ \frac{\text{AUC}\left(x,x\right)-\text{AUC}\left(x^{*b},x^{*b}\left(0\right)\right)}{\text{AUC}\left(x,x\right)-0.5},\text{if }2>\frac{\text{AUC}\left(x,x\right)}{\text{AUC}\left(x^{*b},x^{*b}\left(0\right)\right)}>1,\\ 0,\text{otherwise} & 0. \end{array}\right. \end{array}$$

*(3) Classification Model Construction*. After feature selection, the optimal combination of features was obtained for models of different orders. Imbalance-adjusted bootstrap resampling was performed for all models once more, and the 0.632 + bootstrap AUC of these models was calculated to select the optimal model.

To construct the final prediction model, the coefficients of the optimal combination of features were calculated as follows:

(5) $$\begin{array}{c} {\hat{a}}_{j}=\frac{1}{B}\overset{B}{\underset{b=1}{\sum }}a_{j}\left(x^{*b},y\right),\;\text{for }j=0,1,\ldots ,p, \end{array}$$

where *a*_*j*_(*x*^*∗b*^, *y*) is the coefficient of feature *j* and can be calculated by solving the logistic regression model in equation (1), *p* is the model order, and *j* = 0 refers to the offset of model *y*(*x*_*i*_).

Using the calculated ${\hat{a}}_{j}$, the output of the optimal model *y*(*x*_*i*_) could be obtained by using equation (1), and the final prediction score could be defined as

(6) $$\begin{array}{c} p\left(y_{i}=1\left|x_{i}\right.\right)=\frac{\exp\left[y\left(x_{i}\right)\right]}{1+\exp\left[y\left(x_{i}\right)\right]},\;\text{for }i=1,2,\ldots ,N. \end{array}$$

We employed equation (6) to convert the response of the model into a probabilistic form of output.

### 2.3. Implementation

In this study, all experiments were implemented on a standard personal computer with a single-thread Intel (R) Xeon (R) E5-2667 v4 3.2 GHz processor. The MATLAB 2017b packages used to analyze the radiomics data were available at https://cn.mathworks.com/matlabcentral/fileexchange/51948-radiomics. AlexNet and Inception v3 were pretrained and included in MATLAB 2017b.

Since our dataset was relatively small, the 0.632 + bootstrap resampling method has been used. The principle of this method was to resample all data. For a sample, the selected probability was assumed to be *n*, and the not being selected probability was 1−*n.* For the whole data, if we resampled *n* times, the not being selected probability of a sample was (1 − (1/*n*))^*n*^. When *n* was large enough, lim_*n*⟶*∞*_(1 − (1/*n*))^*n*^ ≈ 1/*e*=0.368 could be obtained, and thus, the selected probability of a sample was 1−0.368 = 0.632. In our experiments, we resampled the original dataset 1000 times and obtained 1000 training sets, so some samples of the original dataset might have appeared multiple times in a training set, and those did not appear eventually formed a validation set. Therefore, after resampling, 1000 pairs of training sets and validation sets could be generated. The number of samples in a training set was 51 × 0.632 ≈ 32, and the number of samples in a validation set was 51 × 0.368 ≈ 19. Classification performance was first evaluated in the training set and then confirmed in the validation set.

## 3. Univariate and Multivariable Results

The *r*_s_ values between the features and glioma recurrence versus necrosis, along with the corresponding *p* values, are listed in Table 3. In the table, only nontexture features and a portion of the texture and deep features are listed. Other detailed *r*_s_ results of handcrafted features are provided in the Supplementary Information. In Table 3, for handcrafted features, such as gray-level run-length matrix (GLRLM)-HGRE and gray-level size zone matrix (GLSZM)-(HGZE, SZLGE, and SZHGE), extracted from the T1C, T2, and FLAIR images, have a slightly higher correlation with glioma recurrence versus necrosis. For deep features, a certain Spearman's rank correlation with glioma necrosis versus recurrence is observed.

**Figure 3:**
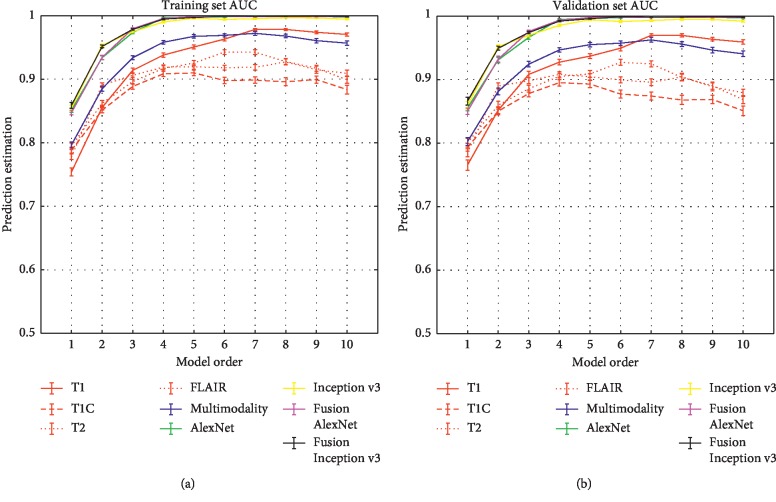
Estimation of the classification performance of multivariable models constructed from T1C, T2, T1, FLAIR, multimodality, AlexNet, Inception v3, fusion AlexNet, and fusion Inception v3 images using optimal features in the training set (a) and validation set (b) for the model orders 1–10. The optimal degrees of freedom were separately found in terms of the maximum 0.632 + bootstrap AUC for each model order. Error bars represent the standard error of the mean at the 95% confidence interval.

Figure 3 shows the prediction performance of the proposed method for the estimation of multivariable models with optimal feature combinations, which were obtained for each model order of the three original feature sets (including T1, T1C, T2, FLAIR, multimodality, AlexNet, and Inception v3 feature sets) and the two fusion feature sets (including fusion AlexNet and fusion Inception v3 feature sets). In Figure 3, the multimodality handcrafted features yielded the highest AUC of 0.9624, sensitivity of 0.8497, and specificity of 0.9083 in model 7 of validation set compared to the single-modality handcrafted features (paired *t*-test, *p* < 0.05, except sensitivity). Model 6 with six features (four handcrafted and two AlexNet features) yielded the highest AUC of 0.9982 in the validation set. Details of the classification accuracy of the optimal model on the training and validation sets are shown in Table 4. The selected features and response map of the optimal model for each feature set are given in the Supplemental Information.

## 4. Discussion

In this study, we proposed a novel radiomics model that is expected to support the classification of glioma recurrence versus necrosis. In the proposed method, MRI images of four modalities (i.e., T1, T2, T1C, and FLAIR images) are used to extract handcrafted and deep features. Fifty-one cases of glioma necrosis and recurrence were applied to validate the proposed method. More importantly, we have obtained the highest classification accuracy on the validation set by using fusion AlexNet features from the perspective of classification accuracy and interpretability of features. Therefore, the proposed method might be a valuable tool for distinguishing glioma necrosis from recurrence.

We employed radiomics to distinguish glioma necrosis from recurrence. We first evaluated the performance of handcrafted features extracted from multimodality and single-modality MRI images. Table 4 shows that the classification accuracy of multimodality MRI is higher than that of single-modality MRI (paired *t*-test *p* < 0.05, except for AUC and sensitivity in T1 modality), which reveals the usefulness of employing different MRI modalities for the current task. However, the extraction methods for handcrafted features are similar for different types of lesions [31–36] and, thus, may limit the potential of radiomics [11]. In order to better reflect the heterogeneity of tumors and improve the performance of radiomics, some scholars have proposed the use of deep features. Antropova et al. [13] used the VGG19 model trained on natural images to extract the deep features of breast cancer and combined it with handcrafted features for the classification of breast cancer, and the results were greatly improved compared with handcrafted features; Decuyper et al. [12] used the trained VGG11 network to extract deep features for different inputs (ROI or the whole image) and classify the grade of glioma into two categories. In addition, Oikonomou et al. [8] show that the combination of handcrafted and deep features leads to the highest performance in lung cancer survival prediction using Random Forest and Naive Bayes classifiers. This successful application of deep features [12–14] confirms the validity of using deep features in our study.

We employed two CNNs (AlexNet and Inception v3) to extract deep features from multimodality MRI images to evaluate the effectiveness of these images for the classification work. In this study, we only extracted features from a given layer of a CNN rather than fine-tuning or training from scratch. Doing so can save computational time [13] and avoid the difficulty in designing CNN. Table 4 reveals that the classification accuracy of using AlexNet and Inception v3 features is higher than that of employing handcrafted features (paired *t*-test *p* < 0.0001). This finding further illustrates the usefulness of deep features in the classification of glioma necrosis versus recurrence. Table 4 also demonstrates that the classification accuracy of using AlexNet features is higher than that of using Inception v3 features (paired *t*-test *p* < 0.0001). The high performance of AlexNet may be due to its simple structure. Complex networks are designed for a specific task; thus, the generalized performance of a complex network is poorer than that of a simple network [13, 37]. Regardless of the performance of deep features, they are less interpretable than handcrafted features, which include tumor shape, volume, texture, and other descriptive features. Therefore, combining deep and handcrafted features provides more information on the object of interest. Finally, we built a six-order model based on the combination of AlexNet and multimodality handcrafted features, ultimately obtaining an AUC of 0.9982, a sensitivity of 0.9941, and a specificity of 0.9755.

Table 5 shows that the classification results of the proposed method outperform the results of recently published papers. Here, only the results of different methods in the literature are listed. Direct comparison of the performances of different methods is unreasonable because various datasets and methods for extracting features and building classifiers are used among studies. Nonetheless, the proposed method shows the highest AUC, specificity, and accuracy among the methods surveyed for the classification problem, thereby implying its advantage for classifying glioma recurrence and necrosis.

**Figure 4:**
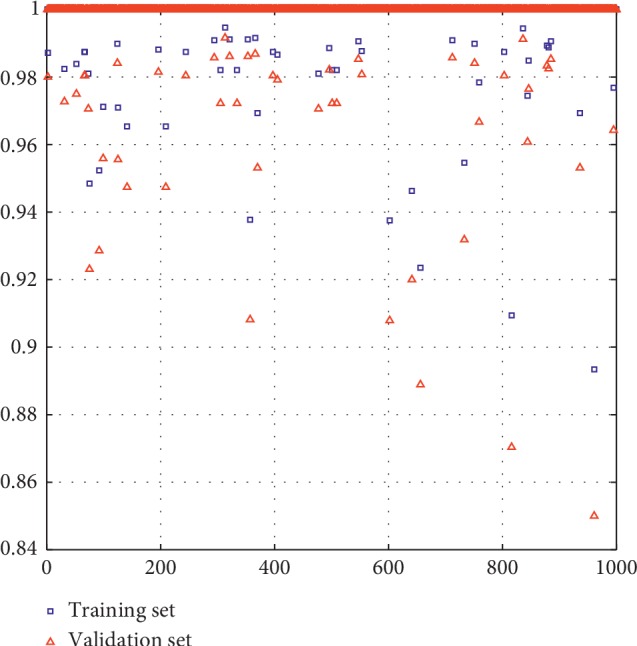
The AUC values of 1000 pairs of the training and validation sets in the classification of glioma necrosis versus recurrence. The *x*-axis represented the number of the experiments and the *y*-axis was the AUC values of the training and validation sets, respectively, measured by each experiment.

The proposed method presents certain limitations. First, the correlations among features were ignored. Despite finding that these correlations could contribute to the model, high-dimensional features were, in general, difficult to handle. Second, there were tens of thousands of features. However, these high-dimensional features were reduced by a stepwise forward feature selection scheme that reduces each of the initial feature sets to only 25 different features through the gain equation (2). Third, the dataset used in this study was relatively small, which was also the problem identified in previous studies [1, 38–42]. Therefore, a large patient cohort was necessary to create a more robust model. In this study, the bootstrap resampling method was used due to the small size of the dataset. We first resampled the whole sample 1000 times and then got 1000 training and validation sets, respectively. For each run of the 1000 experiments, we measured the AUC, sensitivity, and specificity for the training and the validation sets, respectively. Results show that all measurements of the training and the validation sets for each experiment were above 0.8 after combining the deep and the handcrafted features. In order to intuitively show the classification results of glioma necrosis versus recurrence, we used six-order AlexNet deep features and handcrafted features to conduct 1000 experiments as an example.  Figure 4 shows the AUC values of the classification of glioma necrosis versus recurrence. The *x*-axis represented the number of the experiments, and the *y*-axis was the AUC values of the training and validation sets, respectively, measured by each experiment. It can be seen from Figure 4 that the variation tendency of the AUC of the validation sets was the same as that of the training sets, and the difference between the AUC values of the training and validation sets was very small, which indicated that overfitting on the proposed method was alleviated in this study.

In conclusion, finding a noninvasive and accurate method to classify glioma recurrence versus necrosis is clinically significant. In this study, we explored a novel method by combining deep and handcrafted features extracted from multimodality MRI images to improve the classification accuracy of glioma recurrence versus necrosis. Classification models based on objective and quantitative handcrafted and deep features can be useful for precision medicine and improve the treatment strategies used for glioma necrosis and recurrence.

## Figures and Tables

**Table 1:** Clinical characteristics of glioma patients.

Characteristic	Type	Value
Sex	Male	24 (47%)
	Female	29 (53%)
Age	Mean	47.6 (10–74)
Histology	Glioblastoma	12 (23.5%)
	Astrocytoma	14 (27.5%)
	Ependymoma	3 (5.9%)
	Mixed glioma	22 (43.1%)
Grade	High (III-IV)	32 (62.7%)
	Low (I-II)	19 (37.3)
Recurrence or necrosis	Recurrence	35 (68.6%)
	Necrosis	16 (31.4%)
Time interval	Mean	1.8 years
Tumor location	Frontal lobe	21 (41.2%)
	Temporal lobe	22 (43.1%)
	Cerebellum	2 (3.9%)
	Occipital lobe	3 (5.9%)
	Parietal lobe	3 (5.9%)

Time interval refers to the time point from first radiotherapy to diagnosis of necrosis or recurrence. The grade corresponds to the pathological outcome of patients' first surgery.

**Table 2:** MRI protocols for four MRI modalities.

Image	Slice thickness (mm)	TR (ms)	TE (ms)	FA	Matrix	Acquisition time (s)
T2	6	3000	80	90°	376 × 269	72
T1	6	2000	20	90°	284 × 184	102
T1C	6	250	4.6	80°	332 × 246	79.5
FLAIR	6	11000	125	90°	288 × 149	88

**Table 3:** *r*_*s*_ between features (portion of the handcrafted and deep features) and glioma recurrence versus necrosis (*p*=*α*/*K*, *α* = 0.05, and *K* = 176, 4,096, and 2,048 for handcrafted, AlexNet, and Inception v3 features, respectively).

Type	Feature	Modality	*r*_s_	*p* value
Nontexture	Volume	T1	0.0373	0.7949
		T2		
		FLAIR		
		T1C		
	Size	T1	0.0172	0.9045
		T2		
		FLAIR		
		T1C		
	Solidity	T1	0.0115	0.9363
		T2		
		FLAIR		
		T1C		
	Eccentricity	T1	−0.0172	0.9045
		T2		
		FLAIR		
		T1C		
GLRLM	HGRE	T1	0.3273	0.0190
		T2	−0.3331	0.0169
		FLAIR	−0.3187	0.0226
		T1C	0.4594	0.0007
GLSZM	HGZE	T1	0.3790	0.0061
		T2	−0.4508	0.0009
		FLAIR	−0.4852	0.0003
		T1C	0.4738	0.0004
	SZLGE	T1	0.3876	0.0049
		T2	−0.3790	0.0061
		FLAIR	−0.4652	0.0006
		T1C	−0.3962	0.0040
	SZHGE	T1	0.4163	0.0024
		T2	−0.3446	0.0133
		FLAIR	−0.4738	0.0004
		T1C	0.3618	0.0091
AlexNet	F7_618	T1C	0.5656	**0.00001**
	F7_1394	T1	0.5168	0.0001
	F7_2793	FLAIR	0.4823	0.0003
	F7_3501	T2	0.4421	0.0012
Inception v3	avg_pool_663	T1	0.5770	**0.0000093**
	avg_pool__469	T1C	0.5483	0.000031
	avg_pool_827	FLAIR	0.3876	0.005
	avg_pool_774	T2	0.4651	0.000584

For deep feature names, the first character indicates the layer of the CNN and the second character represents the neuron. For example, F7_618 was extracted from a T1C image and taken from the 618th neuron of fully connected layer 7.

**Table 4:** Mean ± standard deviations of the evaluation metrics with different features in the training and validation sets. The results of deep features from each column are shown in bold. The *p* values of paired *t*-tests among different features in the validation set are listed in the lower half of the table. Calculations of the sensitivity and specificity of handcrafted and deep feature sets are provided in the Supplementary Information.

	Training set				Validation set			
Type	AUC	Se	Sp	Acc	AUC	Se	Sp	Acc
FLAIR	0.9429 ± 0.0037	0.7936 ± 0.0129	0.8738 ± 0.0044	0.8598 ± 0.0036	0.9271 ± 0.0047	0.7826 ± 0.0157	0.8421 ± 0.0062	0.8304 ± 0.0052
T1C	0.8980 ± 0.0053	0.6912 ± 0.0117	0.8455 ± 0.0053	0.8094 ± 0.0043	0.8771 ± 0.0065	0.7153 ± 0.0157	0.8032 ± 0.0072	0.7854 ± 0.006
T1	0.9783 ± 0.0017	0.8687 ± 0.0103	0.9284 ± 0.0034	0.9179 ± 0.0029	0.9696 ± 0.0024	0.8529 ± 0.0108	0.9077 ± 0.005	0.8960 ± 0.0043
T2	0.9182 ± 0.0038	0.8109 ± 0.0115	0.8290 ± 0.0044	0.8264 ± 0.0037	0.8994 ± 0.0049	0.8019 ± 0.0144	0.7905 ± 0.0061	0.7909 ± 0.0051
Multimodality	0.9722 ± 0.0029	0.8849 ± 0.0109	0.9190 ± 0.0035	0.9172 ± 0.0033	0.9624 ± 0.0038	0.8497 ± 0.0133	0.9083 ± 0.0052	.8960 ± 0.0047
AlexNet	0.9995 ± 0.0002	0.9996 ± 0.0004	0.9870 ± 0.0015	0.9892 ± 0.0012	**0.9993** ± **0.0003**	**0.9994** ± **0.0006**	**0.9801** ± **0.0022**	**0.9833** ± **0.0018**
Inception v3	0.9941 ± 0.0012	0.9913 ± 0.0034	0.9615 ± 0.0039	0.9669 ± 0.0033	**0.9914** ± **0.0017**	**0.9884** ± **0.0042**	**0.9436** ± **0.0054**	**0.9509** ± **0.0047**
Fusion AlexNet	0.9988 ± 0.0005	0.9957 ± 0.0021	0.9838 ± 0.002	0.9860 ± 0.0017	**0.9982** ± **0.0007**	**0.9941** ± **0.0028**	**0.9755** ± **0.0029**	**0.9786** ± **0.0025**
Fusion Inception v3	0.9992 ± 0.0004	0.9933 ± 0.0025	0.9863 ± 0.0019	0.9874 ± 0.0017	**0.9988** ± **0.0006**	**0.9907** ± **0.0034**	**0.9793** ± **0.0028**	**0.9809** ± **0.0025**
Single-modality handcrafted features compared to multimodality handcrafted features (*p* values)								
T1	—	—	—	—	5.35 × 10^−39^	1.71 × 10^−27^	1.56 × 10^−28^	6.93 × 10^−59^
T2	—	—	—	—	3.57 × 10^−16^	0.1832	6.0 × 10^−26^	1.22 × 10^−22^
T1C	—	—	—	—	3.10 × 10^−23^	0.02	9.71 × 10^−17^	1.68 × 10^−22^
FLAIR	—	—	—	—	0.03	0.4863	0.02	0.0099
Deep features compared to multimodality handcrafted features (*p* values)								
AlexNet	—	—	—	—	3.88 × 10^−138^	6.40 × 10^−117^	8.37 × 10^−208^	7.99 × 10^−301^
Inception v3	—	—	—	—	3.60 × 10^−99^	1.72 × 10^−103^	3.97 × 10^−98^	1.32 × 10^−162^
Fusion AlexNet	—	—	—	—	1.56 × 10^−134^	5.76 × 10^−109^	1.47 × 10^−193^	5.44 × 10^−277^
Fusion Inception v3	—	—	—	—	2.81 × 10^−135^	1.02 × 10^−102^	2.94 × 10^−190^	1.49 × 10^−269^
AlexNet features compared to Inception v3 and fusion AlexNet features, respectively (*p* values)								
Inception v3	—	—	—	—	1.35 × 10^−18^	8.17 × 10^−8^	8.21 × 10^−31^	2.41 × 10^−33^
Fusion AlexNet	—	—	—	—	0.01	1.11 × 10^−4^	0.08	0.02
Fusion Inception v3 features compared to Inception v3 and fusion AlexNet features (*p* values)								
Inception v3	—	—	—	—	2.18 × 10^−14^	0.88	1.81 × 10^−25^	2.09 × 10^−23^
Fusion AlexNet	—	—	—	—	0.8913	0.03	0.42	0.9663

Se: sensitivity; Sp: specificity; Acc: accuracy. “—” in the table indicates the item was not calculated to correspond to paired *t*-test value in the training set.

**Table 5:** Comparison of the classifying results of glioma necrosis versus recurrence.

	Year	Type	Recurrence/necrosis	AUC	Se	Sp	Acc
Tsuyuguchi et al. [38]	2004	PET	6/5	—	**1.00**	0.6	0.82
Ozsunar et al. [39]	2010	PET/MRI (DSCE-CBV, and ASL)	28/7	—	0.94	—	—
Rani et al. [1]	2018	SPECT/MRI (T1, T2, FLAIR, and DWI)	18/10	—	0.92	0.92	—
Takenaka et al. [40]	2014	PET	34/16	0.925	0.912	0.875	—
Jena et al. [41]	2016	PET/MRI (FLAIR, T2, DWI, MRS, and EPI)	19/7	—	—	—	0.97
Jena et al. [42]	2017	PET/MRI (T1, T2, FLAIR, DWI, PWI/EPI, and MRS)	25/10	0.935	—	—	—
Fusion AlexNet		MRI (T1, T2, T1C, and FLAIR)	35/16	**0.9982**	0.9941	**0.9755**	**0.9786**

Se: sensitivity; Sp: specificity; Acc: accuracy; DSCE-CBV: dynamic susceptibility contrast-enhanced cerebral blood volume; ASL: arterial spin-labeled; DWI: diffusion-weighted imaging; EPI: perfusion EPI; PWI: perfusion-weighted imaging; MRS: magnetic resonance spectroscopy.

## Data Availability

The data used to support the findings of this study are available from the corresponding author upon request.

## Abbreviations

T1C: T1-weighted postcontrast
T1: T1-weighted
T2: T2-weighted
FLAIR: Fluid-attenuated inversion recovery
GLRLM: Gray-level run-length matrix
GLSZM: Gray-level size zone matrix
GLCM: Gray-level co-occurrence matrix
NGTDM: Neighbourhood gray-tone difference matrix 0.623 + bootstrap method and the area under the curve metric: 0.632 + bootstrap AUC

## Supplementary Materials

Supplementary Materials Supplementary Material 1: methodology used to extract handcrafted features and texture extraction parameters. Supplementary Material 2: outline of the AlexNet and Inception v3 network architectures. Supplementary Material 3: Figures and Tables of experimental results.

## References

[1] Rani N., Singh B., Kumar N. (2018). Differentiation of recurrent/residual glioma from radiation necrosis using semi quantitative 99mTc MDM (Bis-Methionine-DTPA) brain SPECT/CT and dynamic susceptibility contrast-enhanced MR perfusion. *Clinical Nuclear Medicine*.

[2] Razek A. A. K. A., El-Serougy L., Abdelsalam M., Gaballa G., Talaat M. (2018). Differentiation of residual/recurrent gliomas from postradiation necrosis with arterial spin labeling and diffusion tensor magnetic resonance imaging-derived metrics. *Neuroradiology*.

[3] Stupp R., Roila F. (2009). Malignant glioma: ESMO clinical recommendations for diagnosis, treatment and follow-up. *Annals of Oncology*.

[4] Alexiou G. A., Tsiouris S., Kyritsis A. P., Voulgaris S., Argyropoulou M. I., Fotopoulos A. D. (2009). Glioma recurrence versus radiation necrosis: accuracy of current imaging modalities. *Journal of Neuro-Oncology*.

[5] Shah A. H., Snelling B., Bregy A. (2013). Discriminating radiation necrosis from tumor progression in gliomas: a systematic review what is the best imaging modality?. *Journal of Neuro-Oncology*.

[6] Glantz M. J., Burger P. C., Friedman A. H., Radtke R. A., Massey E. W., Schold S. C. (1994). Treatment of radiation-induced nervous system injury with heparin and warfarin. *Neurology*.

[7] Chuba P. J., Aronin P., Bhambhani K. (1997). Hyperbaric oxygen therapy for radiation-induced brain injury in children. *Cancer*.

[8] Oikonomou A., Khalvati F., Tyrrell P. N. (2018). Radiomics analysis at PET/CT contributes to prognosis of recurrence and survival in lung cancer treated with stereotactic body radiotherapy. *Scientific Reports*.

[9] Lohmann P., Kocher M., Ceccon G. (2018). Combined FET PET/MRI radiomics differentiates radiation injury from recurrent brain metastasis. *NeuroImage: Clinical*.

[10] Zhang Z., Yang J., Ho A. (2018). A predictive model for distinguishing radiation necrosis from tumour progression after gamma knife radiosurgery based on radiomic features from MR images. *European Radiology*.

[11] Lao J., Chen Y., Li Z.-C. (2017). A deep learning-based radiomics model for prediction of survival in glioblastoma multiforme. *Scientific Reports*.

[12] Decuyper M., Bonte S., Van Holen R. (2018). Binary glioma grading: radiomics versus pre-trained CNN features. *Medical Image Computing and Computer Assisted Intervention*.

[13] Antropova N., Huynh B. Q., Giger M. L. (2017). A deep feature fusion methodology for breast cancer diagnosis demonstrated on three imaging modality datasets. *Medical Physics*.

[14] Paul R., Hawkins S. H., Schabath M. B., Gillies R. J., Hall L. O., Goldgof D. B. (2018). Predicting malignant nodules by fusing deep features with classical radiomics features. *Journal of Medical Imaging*.

[15] Paul R., Hawkins S. H., Balagurunathan Y. (2016). Deep feature transfer learning in combination with traditional features predicts survival among patients with lung adenocarcinoma. *Tomography*.

[16] Larsen V. A., Simonsen H. J., Law I., Larsson H. B. W., Hansen A. E. (2013). Evaluation of dynamic contrast-enhanced T1-weighted perfusion MRI in the differentiation of tumor recurrence from radiation necrosis. *Neuroradiology*.

[17] Zhang H., Ma L., Wang Q., Zheng X., Wu C., Xu B.-n. (2014). Role of magnetic resonance spectroscopy for the differentiation of recurrent glioma from radiation necrosis: a systematic review and meta-analysis. *European Journal of Radiology*.

[18] Goenka A. H., Kumar A., Sharma R. (2009). MR spectroscopy for differentiation of recurrent glioma from radiation-induced changes. *American Journal of Roentgenology*.

[19] Reddy K., Westerly D., Chen C. (2013). MRI patterns of T1 enhancing radiation necrosis versus tumour recurrence in high-grade gliomas. *Journal of Medical Imaging and Radiation Oncology*.

[20] Asao C., Korogi Y., Kitajima M. (2005). Diffusion-weighted imaging of radiation-induced brain injury for differentiation from tumor recurrence. *American Journal of Neuroradiology*.

[21] Verma R., Zacharaki E. I., Ou Y. (2008). Multiparametric tissue characterization of brain neoplasms and their recurrence using pattern classification of MR images. *Academic Radiology*.

[22] Huang M., Yang W., Wu Y., Jiang J., Chen W., Feng Q. (2014). Brain tumor segmentation based on local independent projection-based classification. *IEEE Transactions on Biomedical Engineering*.

[23] Di Costanzo A., Scarabino T., Trojsi F. (2006). Multiparametric 3T MR approach to the assessment of cerebral gliomas: tumor extent and malignancy. *Neuroradiology*.

[24] Mouthuy N., Cosnard G., Abarca-Quinones J., Michoux N. (2012). Multiparametric magnetic resonance imaging to differentiate high-grade gliomas and brain metastases. *Journal of Neuroradiology*.

[25] Li Z., Wang Y., Yu J., Guo Y., Cao W. (2017). Deep learning based radiomics (DLR) and its usage in noninvasive IDH1 prediction for low grade glioma. *Scientific Reports*.

[26] Krizhevsky A., Sutskever I., Hinton G. E. (2017). ImageNet classification with deep convolutional neural networks. *Communications of the ACM*.

[27] Szegedy C., Vanhoucke V., Ioffe S., Shlens J., Wojna Z. (2015). Rethinking the inception architecture for computer vision. computer vision and pattern recognition. https://arxiv.org/abs/1512.00567.

[28] Ning Z., Luo J., Li Y. (2019). Pattern classification for gastrointestinal stromal tumors by integration of radiomics and deep convolutional features. *IEEE Journal of Biomedical and Health Informatics*.

[29] Fu L., Ma J., Ren Y., Han Y. S., Zhao J. Automatic detection of lung nodules: false positive reduction using convolution neural networks and handcrafted features.

[30] Reshef D. N., Reshef Y. A., Finucane H. K. (2011). Detecting novel associations in large data sets. *Science*.

[31] Amadasun M. K. R. (1989). Textural features corresponding to textural properties. *IEEE Transactions on Systems, Man, and Cybernetics*.

[32] Chu A., Sehgal C. M., Greenleaf J. F. (1990). Use of gray value distribution of run lengths for texture analysis. *Pattern Recognition Letters*.

[33] Dasarathy B. V., Holder E. B. (1991). Image characterizations based on joint gray level-run length distributions. *Pattern Recognition Letters*.

[34] Galloway M. M. (1975). Texture analysis using gray level run lengths. *Computer Graphics and Image Processing*.

[35] Haralick R. M., Shanmugam K., Dinstein I. H. (1973). Textural features for image classification. *IEEE Transactions on Systems, Man, and Cybernetics*.

[36] Thibault G., Fertil B., Navarro C. (2013). Shape and texture indexes application to cell nuclei classification. *International Journal of Pattern Recognition and Artificial Intelligence*.

[37] Zheng L. Z. Y., Wang S., Wang J., Tian Q. (2016). Good practice in CNN feature transfer. https://arxiv.org/abs/1604.00133.

[38] Tsuyuguchi N., Takami T., Sunada I. (2004). Methionine positron emission tomography for differentiation of recurrent brain tumor and radiation necrosis after stereotactic radiosurgery -in malignant glioma-. *Annals of Nuclear Medicine*.

[39] Ozsunar Y., Mullins M. E., Kwong K. (2010). Glioma recurrence versus radiation necrosis?. *Academic Radiology*.

[40] Takenaka S., Asano Y., Shinoda J. (2014). Comparison of 11C-methionine, 11C-choline, and 18F-fluorodeoxyglucose-PET for distinguishing glioma recurrence from radiation necrosis. *Neurologia Medico-Chirurgica*.

[41] Jena A., Taneja S., Gambhir A. (2016). Glioma recurrence versus radiation necrosis. *Clinical Nuclear Medicine*.

[42] Jena A., Taneja S., Jha A. (2017). Multiparametric evaluation in differentiating glioma recurrence from treatment-induced necrosis using simultaneous 18F-FDG-PET/MRI: a single-institution retrospective study. *American Journal of Neuroradiology*.

