# Supplementary material for: Differentiation of Recurrence from Radiation Necrosis in Gliomas Based on the Radiomics of Combinational Features and Multimodality MRI Images
Source: Comput Math Methods Med. 2019 Dec 1;2019:2893043. doi: 10.1155/2019/2893043 (PMC6913337; doi:10.1155/2019/2893043)
Supplement: Supplementary Materials — Supplementary Material 1: methodology used to extract handcrafted features and texture extraction parameters. Supplementary Material 2: outline of the AlexNet and Inception v3 network architectures. Supplementary Material 3: Figures and Tables of experimental results. [file 2893043.f1.pdf]

# Supplementary Information

## 1. Methodology used to extract handcrafted features and textured extraction parameters.

### 1.1 Handcrafted feature extraction

The handcrafted features extracted from the tumor region were divided into non-textured and textured features. In total, four non-textured features were drawn from the tumor of each patient: (1) Volume (number of voxels in the tumor region multiplied by the dimension of voxels), (2) size (longest diameter of the tumor region), (3) solidity (ratio of the number of voxels in the tumor region to the number of voxels in the 3D convex hull of the tumor region (smallest polyhedron containing the tumor region), and (4) eccentricity. Note that eccentricity refers to the ellipsoid that best fits the tumor region; it is calculated by $\left[1-\frac{axb}{c^2}\right]^{\frac{1}{2}}$, where $c$ is the longest semi-principal axes of the ellipsoid and $a$ and $b$ are the second and third longest semi-principal axes of the ellipsoid, respectively. The textured features were divided into two parts. The first part is based on the intensity histogram features of the tumor region. A total of three intensity histogram features were drawn, namely, variance, skewness, and kurtosis. The second part is based on the matrix features, and a total of 40 features, including 9 gray-level co-occurrence matrix features, 13 GLRLM features, 13 GLSZM features, and 5 neighborhood gray-tone difference matrix features, were extracted. The 43 textured features are shown in Table 1. In this research, data of images of the four modalities were used. Therefore, 172 (43 features are extracted from each image of each MRI modality ) textured features were involved in this

study.

Table 1. Forty-three textural features.

| Texture type | References | Texture name |
| --- | --- | --- |
| ***Global*** | — | Variance |
| | | Skewness |
| | | Kurtosis |
| **GLCM** (Grey-level co-occurrence matrix) | (Haralick *et al* 1973) | Energy |
| | | Contrast |
| | | Correlation |
| | | Homogeneity |
| | | Variance |
| | | Sum Average |
| | | Entropy |
| | — | Dissimilarity |
| | — | AutoCorrelation |
| **GLRLM** (Grey-level run-length matrix) | (Galloway 1975) | Short Run Emphasis (SRE) |
| | | Long Run Emphasis (LRE) |
| | | Grey-Level Non-uniformity (GLN) |
| | | Run-Length Non-uniformity (RLN) |
| | | Run Percentage (RP) |
| | (Chu *et al* 1990) | Low Grey-Level Run Emphasis (LGRE) |
| | | High Grey-Level Run Emphasis (HGRE) |

| | | |
|---|---|---|
| | (Dassarathy and Holder 1991) | Short Run Low Grey-Level Emphasis (SRLGE)<br>Short Run High Grey-Level Emphasis (SRHGE)<br>Long Run Low Grey-Level Emphasis (LRLGE)<br>Long Run High Grey-Level Emphasis (LRHGE) |
| | (Thibault et al 2009) | Grey-Level Variance (GLV)<br>Run-Length Variance (RLV) |
| **GLSZM** (Grey-level size zone matrix) | (Galloway 1975, Thibault *et al* 2009) | Small Zone Emphasis (SZE)<br>Large Zone Emphasis (LZE)<br>Grey-Level Non-uniformity (GLN)<br>Zone-Size Non-uniformity (ZSN)<br>Zone Percentage (ZP) |
| | (Chu et al 1990, Thibault *et al* 2009) | Low Grey-Level Zone Emphasis (LGZE)<br>High Grey-Level Zone Emphasis (HGZE) |
| | (Dasarathy and Holder 1991, Thibault *et al* 2009) | Small Zone Low Grey-Level Emphasis (SZLGE)<br>Small Zone High Grey-Level Emphasis (SZHGE)<br>Large Zone Low Grey-Level Emphasis |

| | | |
|---|---|---|
| | | (LZLGE) |
| | | Large Zone High Grey-Level Emphasis (LZHGE) |
| | (Thibault *et al* 2009) | Grey-Level Variance (GLV)<br>Zone-Size Variance (ZSV) |
| **NGTDM** (Neighbourhood grey-tone difference matrix) | (Amadasun and King 1989) | Coarseness<br>Contrast<br>Busyness<br>Complexity<br>Strength |

## 1.2 Textured feature extraction parameters

In this study, the influence of the following extraction parameters on the predictive value of textures was investigated.

(1) **Wavelet band-pass filtering:** The wavelet basis function 'sym8' in matlab was used in our study. The wavelet basis function first decomposes the tumor region into low-frequency sub-bands (LLL), band-pass sub-bands (LHL, LHH, LLH, HLL, HHL and HLH) and high-frequency sub-bands (HHH). Different weights ($\frac{1}{2}, \frac{2}{3}, 1, \frac{3}{2}$, or 2 ) were used to the eight different frequency sub-bands. The eight sub-bands with different weights were then formed a full frequency tumor region images through inverse wavelet transform. The purpose of this operation was to highlight the tumor information of different frequency sub-bands and help the local preservation of spatial characteristics of tumor region. We used $R$ to denote the weight value, and used $N_R$ to denote the number of the $R$ values, and thus $N_R = 5$.

(2) **Isotropic voxel size:** Before computing the textured features, the neighbouring properties of voxels had to be considered. All volumes were resampled to an isotropic voxel size set to a desired resolution using cubic interpolation. The isotropic voxel size that determined the resolution was denoted as "Scale." The scale values of 1mm, 2mm, 3mm, 4mm, 5mm, and initial in-plane resolution (denoted as "in-pR") were tested. For example, the voxel size of an MRI volume is 0.45mm × 0.45mm × 7mm. If the scale is set to 5 mm, the voxel size could be isotropically resampled to 5mm × 5mm × 5mm. We used $N_{scale}$ to denote the number of the scale values, and thus $N_{scale} = 6$.

(3) **Gray-level quantization:** The full intensity range of the tumor region was quantized to a small number of grey levels. Two extraction parameters were related to the quantization :

**(i) Quantitative algorithms:** Two quantitative algorithms (equal-probability and Lloyd-Max) were implemented using the *histeq* and *lloyds* functions of MATLAB. We used $N_{algo}$ to denote the number of quantitative algorithms, and thus $N_{algo} = 2$.

**(ii) Number of gray levels:** Number of gray levels in the quantized volume was tested on 8, 16, 32, and 64. We used $N_{gl}$ to denote the number of gray levels, and thus $N_{gl} = 4$.

Considering the full set of texture extraction parameters, there are 240 combinations $\left(N_R \times N_{scale} \times N_{algo} \times N_{gl} = 5\times 6\times 2\times 4 = 240\right)$. Under these combinations of parameters, 10,320 textured parameter features were extracted from single-modality MRI images (43 textured features and each textured feature has 240 textured combination parameters, $43\times 240 = 10320$), and 41,280 textured parameter features were drawn from multi-modalities (T1, T1C, T2, and T2-FLAIR) MRI images.

The feature reduction and selection processes were repeated for all possible combinations of textured extraction parameter types, thereby allowing variation. Thus, for experiments in which specific extraction parameters were not allowed to vary,

baseline parameters were defined. In this study, the baseline textured extraction parameters used for the four different types of scans were $R$ = 1, scale = in-pR, algo = Lloyd, and $N$ = 32.

## 2. Outline of the AlexNet and Inception v3 network architectures as shown in Tables 2 and 3, respectively.

Table 2. Outline of the AlexNet network architecture. The tumor region is used as the input. Features are extracted from the second full connection layer (FC7).

| Type | Input | Output |
|---|---|---|
| Input | 3 × 227 × 227 | 3 × 227 × 227 |
| Convolution | 3 × 227 × 227 | 96 × 55 × 55 |
| Max pooling | 96 × 55 × 55 | 96 × 27 × 27 |
| Convolution | 96 × 27 × 27 | 256 × 27 × 27 |
| Max pooling | 256 × 27 × 27 | 256 × 13 × 13 |
| Convolution | 256 × 13 × 13 | 384 × 13 × 13 |
| Convolution | 384 × 13 × 13 | 384 × 13 × 13 |
| Convolution | 384 × 13 × 13 | 256 × 13 × 13 |
| Max pooling | 256 × 13 × 13 | 256 × 6 × 6 |
| Fully connected (FC6) | 256 × 6 × 6 | 4096 × 1 |
| Fully connected (FC7) | 4096 × 1 | 4096 × 1 |
| Fully connected (FC8) | 4096 × 1 | 1000 × 1 |

Table 3. Outline of the Inception v3 network architecture. The tumor region is used as the input. Features are drawn from the avg_pool layer.

| Type | Input | Output |
|---|---|---|
| Input | 3 × 299 × 299 | 3 × 299 × 299 |
| Conv | 3 × 299 × 299 | 32 × 149 × 149 |
| Conv | 32 × 149 × 149 | 32 × 147 × 147 |
| Conv padded | 32 × 147 × 147 | 64 × 147 × 147 |
| Pool | 64 × 147 × 147 | 64 × 73 × 73 |
| Conv | 64 × 73 × 73 | 80 × 71 × 71 |
| Conv | 80 × 71 × 71 | 192 × 35 × 35 |
| Conv | 192 × 35 × 35 | 288 × 35 × 35 |
| 3 × Inception | 288 × 35 × 35 | 768 × 17 × 17 |
| 5 × Inception | 768 × 17 × 17 | 1280 × 8 × 8 |
| 2 × Inception | 1280 × 8 × 8 | 2048 × 8 × 8 |
| Avg_pool | 2048 × 8 × 8 | 2048 × 1 × 1 |
| Linear | 2048 × 1 × 1 | 1000 × 1 × 1 |
| Softmax | 1000 × 1 × 1 | 1000 × 1 |

## 3. The sensitivity and specificity of handcrafted and deep feature sets can be defined as follows:

$$\left[\hat{S}\right]_{0.632+} = \frac{1}{B}\sum_{b=1}^{B}\left[\left(1-\alpha(b)\right)\cdot S(x,x)+\alpha(b)\cdot S\left(x^{*b},x^{*b}(0)\right)\right]$$

where $$\alpha(b) = \frac{0.632}{1-0.368\cdot R(b)}$$

and $$R(b)=\begin{cases}\dfrac{S(x,x)-S\left(x^{*b},x^{*b}(0)\right)}{S(x,x)} & \text{if } \dfrac{S(x,x)}{S\left(x^{*b},x^{*b}(0)\right)}>1\\ 0 & \text{otherwise,}\end{cases}$$

for S:Sensitivity, Specificity

**4. Table 4 presents the $r_s$ between handcrafted features and glioma recurrence versus necrosis, along with their corrsponding *p* values.**

| Type | Feature | Modality | $r_s$ | *P* value |
|---|---|---|---|---|
| Non-Texture | Volume | T1 | | |
| | | T2 | 0.0373 | 0.7949 |
| | | FLAIR | | |
| | | T1C | | |
| | Size | T1 | | |
| | | T2 | 0.0172 | 0.9045 |
| | | FLAIR | | |
| | | T1C | | |
| | Solidity | T1 | | |
| | | T2 | 0.0115 | 0.9363 |
| | | FLAIR | | |
| | | T1C | | |
| | Eccentricity | T1 | | |
| | | T2 | -0.0172 | 0.9045 |
| | | FLAIR | | |
| | | T1C | | |

| | | | | |
|---|---|---|---|---|
| Global | Variance | T1 | -0.2067 | 0.1455 |
| | | T2 | -0.2240 | 0.1141 |
| | | FLAIR | -0.1034 | 0.4704 |
| | | T1C | -0.1608 | 0.2597 |
| | Skewness | T1 | -0.0689 | 0.6309 |
| | | T2 | -0.2326 | 0.1005 |
| | | FLAIR | 0.3158 | 0.0240 |
| | | T1C | 0.2240 | 0.1141 |
| | Kurtosis | T1 | 0.1378 | 0.3348 |
| | | T2 | 0.2613 | 0.0640 |
| | | FLAIR | 0.1866 | 0.1897 |
| | | T1C | -0.2383 | 0.0922 |
| LCM | Energy | T1 | -0.2756 | 0.0503 |
| | | T2 | -0.3532 | 0.0110 |
| | | FLAIR | -0.2900 | 0.0390 |
| | | T1C | -0.3761 | 0.0065 |
| | Contrast | T1 | 0.2986 | 0.0333 |
| | | T2 | 0.3101 | 0.0268 |
| | | FLAIR | -0.1694 | 0.2347 |
| | | T1C | -0.3187 | 0.0226 |
| | Entropy | T1 | 0.2900 | 0.0390 |
| | | T2 | 0.3388 | 0.0150 |

| | | | |
|---|---|---|---|
| | FLAIR | 0.2412 | 0.0882 |
| | T1C | 0.3962 | 0.0040 |
| Homogeneity | T1 | -0.2642 | 0.0610 |
| | T2 | 0.2699 | 0.0554 |
| | FLAIR | -0.1292 | 0.3662 |
| | T1C | -0.2441 | 0.0844 |
| Correlation | T1 | -0.2756 | 0.0503 |
| | T2 | -0.2240 | 0.1141 |
| | FLAIR | 0.1378 | 0.3348 |
| | T1C | 0.1120 | 0.4340 |
| Sum Average | T1 | -0.2326 | 0.1005 |
| | T2 | 0.3532 | 0.0110 |
| | FLAIR | -0.3331 | 0.0169 |
| | T1C | -0.4049 | 0.0032 |
| Variance | T1 | 0.2153 | 0.1291 |
| | T2 | -0.2929 | 0.0370 |
| | FLAIR | -0.2986 | 0.0333 |
| | T1C | 0.4020 | 0.0035 |
| Dissimilarity | T1 | 0.2843 | 0.0432 |
| | T2 | 0.3359 | 0.0159 |
| | FLAIR | 0.1751 | 0.2189 |

| | | | | |
|---|---|---|---|---|
| | | T1C | 0.2670 | 0.0582 |
| | Auto Correlaion | T1 | -0.3044 | 0.0299 |
| | | T2 | 0.3417 | 0.0141 |
| | | FLAIR | -0.3647 | 0.0085 |
| | | T1C | -0.4135 | 0.0026 |
| GLRLM | SRE | T1 | -0.2010 | 0.1573 |
| | | T2 | -0.2957 | 0.0351 |
| | | FLAIR | -0.1895 | 0.1829 |
| | | T1C | 0.1952 | 0.1697 |
| | LRE | T1 | -0.2182 | 0.1240 |
| | | T2 | 0.2957 | 0.0351 |
| | | FLAIR | 0.1838 | 0.1968 |
| | | T1C | 0.1694 | 0.2347 |
| | GLN | T1 | 0.1895 | 0.1829 |
| | | T2 | 0.1924 | 0.1762 |
| | | FLAIR | 0.1838 | 0.1968 |
| | | T1C | 0.1838 | 0.1968 |
| | RLN | T1 | 0.2469 | 0.0807 |
| | | T2 | 0.2211 | 0.1190 |
| | | FLAIR | 0.1866 | 0.1897 |
| | | T1C | 0.1952 | 0.1697 |
| | RP | T1 | 0.2297 | 0.1049 |

| | | | |
|---|---|---|---|
| | T2 | -0.3015 | 0.0316 |
| | FLAIR | -0.1924 | 0.1762 |
| | T1C | 0.1780 | 0.2114 |
| LGRE | T1 | -0.3876 | 0.0049 |
| | T2 | -0.4192 | 0.0022 |
| | FLAIR | -0.3647 | 0.0085 |
| | T1C | -0.3876 | 0.0049 |
| HGRE | T1 | 0.3273 | 0.0190 |
| | T2 | -0.3331 | 0.0169 |
| | FLAIR | -0.3187 | 0.0226 |
| | T1C | 0.4594 | 0.0007 |
| SRLGE | T1 | -0.3848 | 0.0053 |
| | T2 | -0.4163 | 0.0024 |
| | FLAIR | -0.3790 | 0.0061 |
| | T1C | -0.3474 | 0.0125 |
| SRHGE | T1 | 0.2814 | 0.0455 |
| | T2 | 0.2814 | 0.0455 |
| | FLAIR | -0.2498 | 0.0771 |
| | T1C | 0.4049 | 0.0032 |
| LRLGE | T1 | -0.3819 | 0.0057 |
| | T2 | -4192 | 0.0022 |
| | FLAIR | 0.3618 | 0.0091 |

| | | | | |
|---|---|---|---|---|
| | | T1C | 0.3130 | 0.0253 |
| | LRHGE | T1 | 0.2010 | 0.1573 |
| | | T2 | 0.3503 | 0.0117 |
| | | FLAIR | -0.2527 | 0.0736 |
| | | T1C | -0.3331 | 0.0169 |
| | GLV | T1 | -0.2354 | 0.0963 |
| | | T2 | -2756 | 0.0503 |
| | | FLAIR | -0.3187 | 0.0226 |
| | | T1C | -0.3158 | 0.0240 |
| | RLV | T1 | -0.3158 | 0.0240 |
| | | T2 | -2871 | 0.0411 |
| | | FLAIR | -0.3072 | 0.0283 |
| | | T1C | -0.3245 | 0.0202 |
| GLSZM | SZE | T1 | 0.4135 | 0.0026 |
| | | T2 | -3176 | 0.0065 |
| | | FLAIR | -0.3790 | 0.0061 |
| | | T1C | -0.3733 | 0.0070 |
| | LZE | T1 | 0.2096 | 0.1399 |
| | | T2 | 0.2871 | 0.0411 |
| | | FLAIR | 0.2441 | 0.0844 |
| | | T1C | 0.1694 | 0.2347 |
| | GLN | T1 | 0.3216 | 0.0214 |

| | | | |
|---|---|---|---|
| | T2 | 0.2785 | 0.0478 |
| | FLAIR | 0.1924 | 0.1762 |
| | T1C | 0.2182 | 0.1240 |
| ZSN | T1 | 0.3589 | 0.0097 |
| | T2 | 0.2527 | 0.0736 |
| | FLAIR | 0.1809 | 0.2040 |
| | T1C | 0.2613 | 0.0640 |
| ZP | T1 | -0.2498 | 0.0771 |
| | T2 | -0.2900 | 0.0390 |
| | FLAIR | -0.3072 | 0.0283 |
| | T1C | 0.2584 | 0.0671 |
| LGZE | T1 | -0.4020 | 0.0035 |
| | T2 | -0.4192 | 0.0022 |
| | FLAIR | -0.4422 | 0.0012 |
| | T1C | -0.3273 | 0.0190 |
| HGZE | T1 | 0.3790 | 0.0061 |
| | T2 | -0.4508 | 0.0009 |
| | FLAIR | -0.4852 | 0.0003 |
| | T1C | 0.4738 | 0.0004 |
| SZLGE | T1 | 0.3876 | 0.0049 |
| | T2 | -0.3790 | 0.0061 |
| | FLAIR | -0.4652 | 0.0006 |

| | | | | |
|---|---|---|---|---|
| | | T1C | -0.3962 | 0.0040 |
| | SZHGE | T1 | 0.4163 | 0.0024 |
| | | T2 | -0.3446 | 0.0133 |
| | | FLAIR | -0.4738 | 0.0004 |
| | | T1C | 0.3618 | 0.0091 |
| | LZLGE | T1 | -0.2584 | 0.0671 |
| | | T2 | -0.3618 | 0.0091 |
| | | FLAIR | 0.3072 | 0.0283 |
| | | T1C | -0.2843 | 0.0432 |
| | LZHGE | T1 | 0.2182 | 0.1240 |
| | | T2 | 0.2756 | 0.0503 |
| | | FLAIR | 0.2441 | 0.0844 |
| | | T1C | -0.2240 | 0.1141 |
| | GLV | T1 | -0.2527 | 0.0736 |
| | | T2 | -0.2326 | 0.1005 |
| | | FLAIR | -0.2670 | 0.0582 |
| | | T1C | -0.2010 | 0.1573 |
| | ZSV | T1 | -0.4049 | 0.0032 |
| | | T2 | -0.3503 | 0.0117 |
| | | FLAIR | 0.2642 | 0.0610 |
| | | T1C | -0.3302 | 0.0180 |
| NGTDM | Coarseness | T1 | -0.2326 | 0.1005 |

| | | | |
|---|---|---|---|
| | T2 | -0.2297 | 0.1049 |
| | FLAIR | -0.1924 | 0.1762 |
| | T1C | -0.2039 | 0.1513 |
| Contrast | T1 | 0.3675 | 0.0080 |
| | T2 | 0.3015 | 0.0316 |
| | FLAIR | 0.2699 | 0.0554 |
| | T1C | 0.4192 | 0.0022 |
| Busyness | T1 | 0.2527 | 0.0736 |
| | T2 | 0.2153 | 0.1291 |
| | FLAIR | 0.2900 | 0.0390 |
| | T1C | 0.2441 | 0.0844 |
| Complexity | T1 | 0.3273 | 0.0190 |
| | T2 | 0.3273 | 0.0190 |
| | FLAIR | 0.2469 | 0.0807 |
| | T1C | 0.2555 | 0.0703 |
| Strength | T1 | -0.2642 | 0.0610 |
| | T2 | -0.2153 | 0.1291 |
| | FLAIR | -0.1809 | 0.2040 |
| | T1C | -0.2153 | 0.1291 |

$$p = \frac{\alpha}{K} = \frac{0.05}{176}$$

## 5. Probability of developing glioma recurrent (*R*) or necrotic (*N*) as a function of the response of the multivariable model proposed in this

**work**

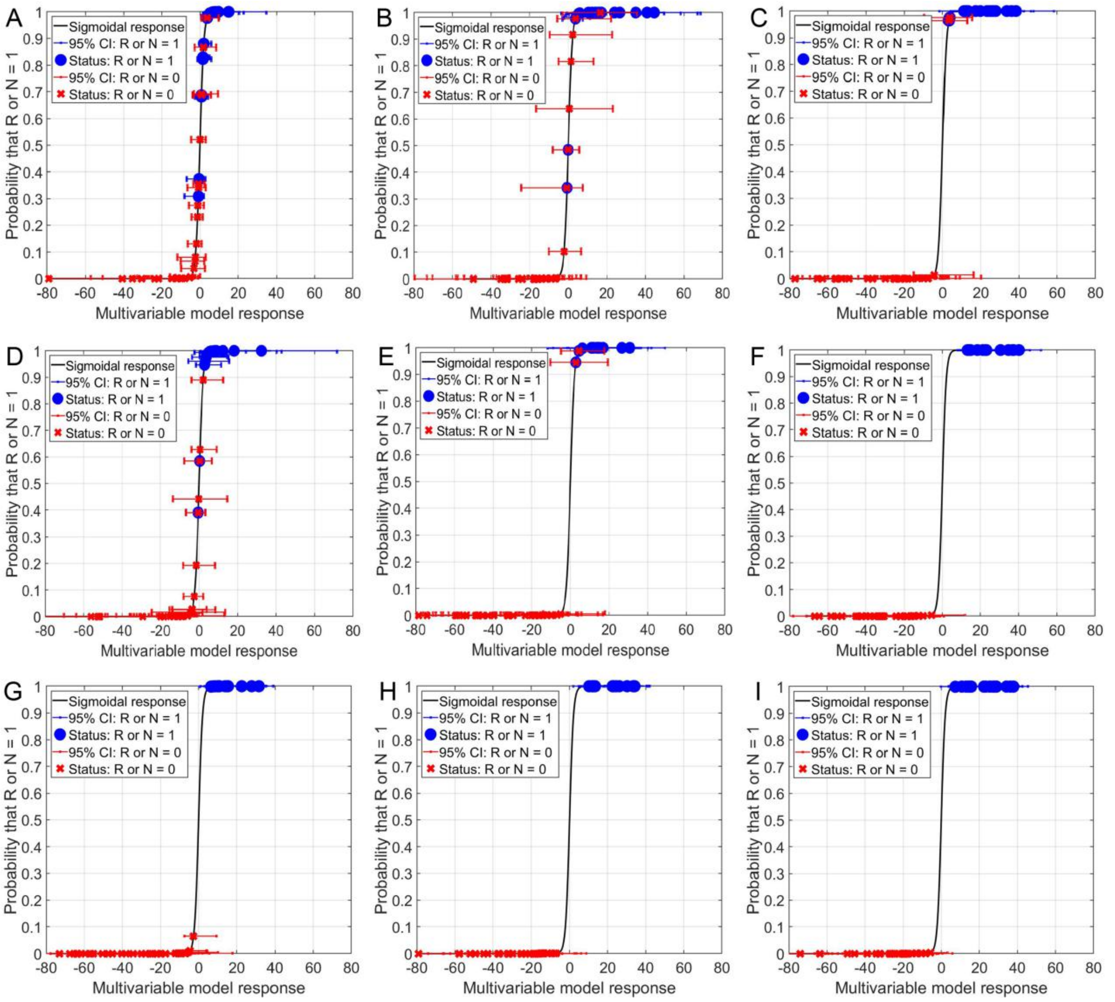


Fig. 1 A–I respectively refer to the responses from T1C, T2, T1, T2-FLAIR, multi-modality, AlexNet, Inception v3, fusion AlexNet, and fusion Inception v3 features. The red crosses represent patients with glioma recurrence, and the blue dots refer to patients with glioma necrosis. The red crosses or blue dots between the two horizontal lines represent patients with uncertainty or misclassification.

## 6. Imaging features of the best model constructed from individual data sets

### Textural features extracted from T1 images

**Model:**

(-170.7) × T1(R=2.00,Scale=5,Quant.algo=Lloyd,Ng=64)--GLSZM-GLV

+ 445.5 × T1(R=1.50,Scale=3,Quant.algo=Lloyd,Ng=8)--GLSZM-SZLGE

+ 4.558 × T1(R=1.50,Scale=4,Quant.algo=Lloyd,Ng=8)--GLSZM-HGZE

+ (-63.22) × T1(R=1.50,Scale=pixelW,Quant.algo=Lloyd,Ng=64)--GLCM-Homogeneity

+ (-0.06402) × T1(R=2.00,Scale=pixelW,Quant.algo=Lloyd,Ng=32)--GLSZM-GLN

+ 689.1 × T1(R=0.67,Scale=pixelW,Quant.algo=Lloyd,Ng=64)--NGTDM-Contrast

+ (-733.6) × T1(R=2.00,Scale=pixelW,Quant.algo=Equal,Ng=16)--GLCM-Correlation

+ 574.2

**Textural features extracted from T1C images**

**Model:**

0.07604 × T1C(R=1.50,Scale=4,Quant.algo=Equal,Ng=64)--GLRLM-HGRE

+ (-0.01174) × T1C(R=2.00,Scale=pixelW,Quant.algo=Lloyd,Ng=64)--GLRLM-LRHGE

+ (-2.078) × T1C(R=0.50,Scale=2,Quant.algo=Equal,Ng=16)--GLCM-AutoCorrelation

+ (-5.527) × T1C(R=1.00,Scale=1,Quant.algo=Equal,Ng=8)--Global-Kurtosis

+ 74.54 × T1C(R=0.67,Scale=3,Quant.algo=Equal,Ng=16)--GLSZM-LGZE

+ 4721 × T1C(R=0.67,Scale=1,Quant.algo=Equal,Ng=16)--GLCM-SumAverage

-60.2

**Textural features extracted from T2 images**

**Model:**

(-1519000) × T2(R=1.50,Scale=pixelW,Quant.algo=Equal,Ng=64)--GLSZM-ZSV

+ (-1.911) × T2(R=0.67,Scale=pixelW,Quant.algo=Equal,Ng=8)--GLSZM-HGZE

+ 7.548 × T2(R=0.50,Scale=5,Quant.algo=Equal,Ng=8)--Global-Kurtosis

+ 0.08183 × T2(R=1.50,Scale=4,Quant.algo=Lloyd,Ng=32)--GLRLM-LRHGE

+ (-3.318) × T2(R=0.67,Scale=pixelW,Quant.algo=Equal,Ng=8)--GLRLM-HGRE

+ 161 × T2(R=2.00,Scale=pixelW,Quant.algo=Equal,Ng=64)--NGTDM-Contrast

+ 45.25

**Textural features extracted from FLAIR images**

**Model:**

29.09 × FLAIR(R=2.00,Scale=4,Quant.algo=Equal,Ng=32)--GLCM-Entropy

+ (-0.5803) × FLAIR(R=1.50,Scale=1,Quant.algo=Equal,Ng=16)--GLSZM-SZHGE

+ (-1125) × FLAIR(R=0.67,Scale=4,Quant.algo=Equal,Ng=64)--GLSZM-SZLGE

+ (-638) × FLAIR(R=0.67,Scale=5,Quant.algo=Equal,Ng=8)--GLCM-Variance

+ 245.5 × FLAIR(R=1.50,Scale=4,Quant.algo=Lloyd,Ng=8)--GLSZM-ZSV

+ 292.7 × FLAIR(R=2.00,Scale=pixelW,Quant.algo=Lloyd,Ng=64)--NGTDM-Contrast

-221.8

**Textural features extracted from multi-modalities images**

**Model:**

247.4 × T1C(R=1.50,Scale=pixelW,Quant.algo=Lloyd,Ng=64)--GLSZM-SZE

+ (-550.8) × T2(R=1.50,Scale=pixelW,Quant.algo=Lloyd,Ng=8)--GLRLM-LGRE

+ (-3.995) × T2(R=0.67,Scale=pixelW,Quant.algo=Equal,Ng=8)--GLSZM-HGZE

+ 2.66 × T1(R=0.50,Scale=pixelW,Quant.algo=Lloyd,Ng=8)--GLSZM-HGZE

+ 253.7 × T1(R=1.50,Scale=pixelW,Quant.algo=Lloyd,Ng=64)--NGTDM-Contrast

+ 14.65 × T2(R=2.00,Scale=pixelW,Quant.algo=Lloyd,Ng=64)--GLCM-Dissimilarity

+ (-0.1769) × FLAIR(R=1.00,Scale=pixelW,Quant.algo=Equal,Ng=32)--GLSZM-SZHGE

-77.43

**Alexnet features extracted from multi-modalities images**

**Model:**

4.834 × T2_F7_3501

+ (-5.983) × T1C_F7_783 + 5.518 × FLAIR_F7_647 + (-7.081) × T2_F7_2703 + 4.844 × T1_F7_1421

+ 6.404 × FLAIR_F7_598

+ 2.477 × T1_F7_1394 + 13.51

**Inceptionv3 features extracted from multi-modalities images**

**Model:**

(-65.41) × T1_avg_pool_1268 + (-124.1) × T2_avg_pool_1732 + (-62.1) × T1C_avg_pool_172 + (-49.23) × T1C_avg_pool_1930 + (-38.36) × T1C_avg_pool_1857 + (-24.96) × T1C_avg_pool_1153 + 37.98

**Fusion alexnet features extracted from multi-modalities images**

**Model:**

(-5.119) × T1C_F7_2133 + (-10.51) × T2_F7_2703 + 5.291 × FLAIR_F7_598 + 4.764 × T1C_F7_2535 + (-1.241) × FLAIR (R=1.50,Scale=1,Quant.algo=Equal,Ng=16)--GLSZM-SZHGE

+ 1.076 × T1(R=1.50,Scale=4,Quant.algo=Lloyd,Ng=8)--GLSZM-HGZE - 11.31

**Fusion inceptionv3 features extracted from multi-modalities images**

**Model:**

(-0.4733) × FLAIR(R=1.00,Scale=pixelW,Quant.algo=Equal,Ng=16)--GLSZM-HGZE + (-152.7) × T1C_avg_pool_1930 + 86.65 × T1C_avg_pool_1759 + 2.819 × T1(R=1.50,Scale=4,Quant.algo=Lloyd,Ng=8)--GLSZM-HGZE

+ (-1181) × FLAIR(R=0.67,Scale=4,Quant.algo=Equal,Ng=64)--GLSZM-SZLGE + (-52.88) ×

T2_avg_pool_1732 - 14.91
